# Effects of ocular hypothermia: potential perspectives in vitreoretinal surgery

**DOI:** 10.1186/s40942-025-00667-4

**Published:** 2025-04-15

**Authors:** Emanuele Crincoli, Matteo Mario Carlà, Alfonso Savastano, Mariacristina Savastano, Raphael Kilian, Clara Rizzo, Tomaso Caporossi, Stanislao Rizzo

**Affiliations:** 1 Ophthalmology Unit, “Fondazione Policlinico Universitario A. Gemelli IRCCS”, Rome, Italy; 2 Catholic University of “Sacro Cuore”, Rome, Italy; 3 Ophthalmology Unit, University of Verona, Verona, Italy; 4 Department of Surgical, Medical and Molecular Pathology and Critical Care Medicine, University of Pisa, Pisa, Italy; 5 Vitreoretinal Surgery Unit, Isola Tiberina Gemelli Isola Hospital, Rome, Italy; 6 “Consiglio Nazionale delle Ricerche, Istituto di Neuroscienze”, Pisa, Italy

**Keywords:** Temperature, Hypothermia, Retina, Retinal pigment epithelium, Ganglion cells, Choroid, Ciliary body, Vitreoretinal surgery, Inflammation

## Abstract

**Purpose:**

To summarize knowledge about the effects of experimental and iatrogenic hypothermia on ocular structures, with a specific focus on retinal consequences and therapeutical perspectives in vitreoretinal surgery.

**Materials and methods:**

This review of the literature includes a section on the effects of low temperature on different ocular structures (sclera, choroid, retina, vitreous and ciliary body), a focus on the effect on retinal pigment epithelium (RPE), retinal neurons and inflammation and a section about results of vitreoretinal surgery performed at low temperature. In vitro, animal and human studies were included.

**Results:**

Temperature changes induce several regulatory responses within the eye, including modifications of intraocular pressure (IOP), local blood flow, cytokine secretion and cellular metabolism. Cooling of retinal structures has been demonstrated to induce beneficial effects including increased survival of RPE and retinal neurons. Vitreoretinal surgery performed at lower intraocular temperatures has shown positive effect on postoperative inflammation, even though the rebound effect of a sudden postoperative temperature increase seems to be detrimental.

**Conclusions:**

Despite being a promising approach, vitreoretinal surgery performed under lower intraocular temperature conditions deserves refinement in its methodologies. Hopefully, new randomized clinical trials will provide indications on how to apply this technique in the safest and most effective way.

## Abstract (significance)

**What is known**

• The direct or indirect influence of temperature changes of even only a few Celsius degrees can result in profound modifications of numerous and various biological processes.

• Moderate hypothermia can block the cascade of destructive inflammation, prevent blood–aqueous barrier disruption and local edema.

**What is new**

• Local moderate hypothermia has been demonstrated to induce reduction of the inflammation and increased cell survival in the eye.

• Rebound hyperthermia control strategies are needed in vitreoretinal surgery to benefit from these positive effects.

## Introduction

The direct or indirect influence of temperature changes of even only a few Celsius degrees can result in profound modifications of numerous and various biological processes [1]. The beneficial effects of controlled moderate hypothermia (33.9–32 °C) have long been used in other areas of medicine, with its main application being increasing resistance of neural and glial cells to ischemic conditions [2]. In fact, moderate hypothermia can block the cascade of destructive inflammation, prevent blood–aqueous barrier disruption and local edema, and possibly stimulate the release of protective proteins [3]. 

However, to ensure these beneficial effects, duration of hypothermia and rate of tissue rewarming should be carefully regulated. In fact, hypothermia results in local reduction of blood flow with associated decrease in enzymatic activity and local hypoxia. Uncontrolled rewarming can thus result in tissue damage similar to the one reported in ischemia-reperfusion and hypoxia-reoxygenation syndrome [4]. Potential beneficial and negative effects have been described in case of use of local hypothermia in the management of ocular conditions, especially in the context of vitreoretinal diseases. The aim of this review is to summarize knowledge about the effects of experimental and iatrogenic hypothermia on ocular structures, with a specific focus on retinal consequences and therapeutical perspectives in vitreoretinal surgery.

### Factors concurring to intraocular temperature variation in natural conditions

Temperature of each component of the eye is influenced by different factors depending on their specific location.

- Body temperature and local blood flow: Proximity to vascular structures increases local temperature up to the limit of body temperature (approximately 37 °C). Local temperature is the closer to body temperature the higher the flow and the slower the flow rate. Basically, a high flow / slow flow-rate vasculature results in maximization of heat exchange between blood and intraocular structures. This condition typically occurs in the choroid, iris and ciliary body, whose local temperature is generally close to body temperature. Elevations of intraocular pressure(IOP) reduce choroidal blood flow leading to an increase in ocular temperature [5]. Detailed analysis of vectorial blood stream in the choroid allowed accurate prediction of local temperature in a model elaborated by Heussner et al. [6]
- Environmental heat dispersion: proximity and width of the surface of contact with the external environment make local temperature closer to room temperature, which is estimated to a standard 22 °C [7]. The outer surface of the cornea is generally maintained at 32–33 °C, but the inner surface of the cornea is at a higher temperature (only 2–4 °C less than the BT due to corneal thermal resistance).However, this small temperature gradient(2–4 °C) across the anterior chamber is believed to be the dominant mechanism driving the fluid flow within it [8].
- Light radiation: Exposure to natural and artificial sources of light radiation can increase local tissue temperature in the eye. As ambient light is constantly focused on the foveal region, a continuous cooling of the retina is essential to maintain its homeostasis, a task which is prevalently accomplished by the choroid [5]. In fact, choroidal flow absorbs and disperses local heat generated by the effect of light. The protective role of choroidal flow is therefore fundamental and it gradually fades away as choroidal flow decreases with aging and pathological conditions [9].

### Effects of temperature on different ocular structures

#### Choroid and sclera

Ocular and periocular temperature variations induce changes in choroidal blood flow. The main role in this regulatory response is attributed to local trigeminal reflex. In fact, sensory innervation of the choroid includes trigeminal fibers from the trigeminal ganglion co-containing substance P (SP) and calcitonin gene-related peptide (CGRP). Nerve fibers reach the choroid travelling with the short ciliary nerves and on blood vessels within the orbit. Sensory fibers can influence their innervation territory in either of two ways – by sending a message centrally to initiate a reflex response, or by direct local release of SP/CGRP in response to activating stimuli [10]. Local hypothermia elicits the release of SP and CGRP with both mechanisms, causing vasodilation and increasing choroidal blood flow as a result [11]. In rabbit retina, this compensatory reflexes are activated by temperatures higher or lower than 34 °C and are able to stabilize retinal temperature during exposure to local temperatures ranging from 30° to 40 °C [12]. Therefore, integrity of choroidal thermoregulation reflex is important to ensure stable temperatures in the retina, which in turn seems to be a crucial factor for preservation of retinal homeostasis and function. Horiguchi et al. [13] demonstrated in vivo electroretinographic alterations in vitrectomized eyes exposed to balanced salt solution at 27–28 °C. In particular, a global reduction of amplitudes and delayed peak times were detected. Nevertheless, these abnormalities completely resolved after cessation of the cooling stimulus.

Cold-sensory trigeminal fibers have also been detected in the sclera and iris, where they are believed to play a role similar to the one of the choroid in blood flow regulation. In an experimental setting, occlusion of the carotid artery for 30 to 60 s significantly increased the firing frequency in almost all analyzed neuronal terminations in the sclera and iris. The firing frequency (corresponding to sensitivity of the regulatory mechanism) was at its highest for temperature ranges between 30° and 35 °C, while below 10 °C impulse firing was markedly reduced. Nevertheless, no firing modification was noted when occlusion was performed in a temperature-controlled setting with the thermode set to 33 °C, meaning that this temperature could represent the equilibrium point of the system. Increased firing frequency resulted in a sudden local blood flow decrease to half of the control value [14]. Another temperature-sensitive mechanism responsible for temperature-induced changes within the choroid involves the activation of the transient receptor potential melastatin 8 (TRPM8) channel. TRPM8 is the most important cold sensor in mammals playing a pivotal role in thermoregulation and energy homeostasis [15]. TRPM8-immunolabelled fibers have been found to surround and travel along choroidal and ciliary body vasculature. Moreover, TRPM8-knock-out animals demonstrated a poor compensatory response to local hypothermia, with a faster decrease in intraocular temperature in response to ocular cooling [16]. 

#### RPE and photoreceptors

Retinal pigment epithelium (RPE) and photoreceptors (PR) are highly specialized cells characterized by a high metabolism and the highest oxygen consumption per unit weight of all human tissues. Reduction of blood flow supplying these cells results in a metabolic unbalance leading to calcium overload, acidosis, free radical generation, nitric oxide overproduction and activation of caspases [17]. As a result, RPE an PR encounter a progressive degeneration and RPE production of vascular endothelial growth factor (VEGF) increases in an attempt to maximize local oxygen supply, leading to choroidal neovascularization [18]. To slow down this disruptive cascade, two different strategies may be adopted: on one hand to reduce RPE’s and PR’s metabolic demand and, on the other hand, to improve local blood flow. Among the first type of strategies, local hypothermia has been proposed as an efficient method to reduce local metabolism in the outer retina and protect RPE and PR against inflammatory damage resulting from hypoxia. Coassin et al. [19] demonstrated in a normoxic setting a significant reduction in cells’ metabolism in ARPE-19 cells cultivated in hypothermic conditions compared to ARPE-19 cells cultivated at 37 °C, with a reduction of -17% and − 31% at 34 °C and 27 °C respectively. They also demonstrated a reduction of -38% in VEGF secretion at 34 °C, also showing the persistence of this effect for 4 days after the cessation of hypothermia. Other authors confirmed the beneficial effect of hypothermia on VEGF A production in ARPE-19 cells, demonstrating also the persistent production of protective pigment epithelium-derived factor (PEDF) at the same temperature [20]. Moreover, RNA-binding motif protein 3 (RBM3) and cold inducible RNA-binding protein (CIRP) are cold-shock proteins (CSPs) that are rapidly produced in mammalian retina when RPE-like cells are exposed to a temperature of 32 °C [21]. RBM3 is a crucial factor which mediates hypothermia-induced neuroprotective effects by inhibiting the mitogen-activated protein kinase (MAPK) signaling of p38, JNK, and ERK. CIRBP is a RNA-binding factor enhancing expression of TRX, an important ROS scavenger that helps prevent caspase activation and therefore apoptosis [22]. 

Hypothermia has also demonstrated beneficial effects in induced pluripotent stem cells -derived RPE (hiPSC-RPE). These cell sheets can be used for autologous hiPSC-derived transplants in the context of age-related macular degeneration(AMD) [23]. These cells were found to be highly sensible to temperature increase, with a significant higher rate of cell loss at body temperature compared with 16 °C and 22 °C temperature [24]. 

#### Muller cells and other retinal cells

Mild hypothermia (32 °C) was shown to protect Muller cells and ganglion cells(GCs) from ischemia and ischemia-reperfusion(I/R)-related damage. In fact, ocular hypothermia significantly prevented the decrease in the electroretinogram (ERG) a-wave, b-wave, and oscillatory potentials (OPs) amplitude induced by I/R. The protective effect of ocular hypothermia was also evident at histological level since it significantly prevented the decrease in total retinal, inner nuclear layer (INL), and inner plexiform layer (IPL) thickness, and the number of Brn3a(+) cells in the ganglion cell layer (GCL) induced by 40- min ischemia. However, ocular hypothermia did not affect the decrease in ONL thickness induced by I/R.^25^ Other authors demonstrated in an animal model of perinatal asphyxia that hypothermia may prevent inner retinal layers thickness increase and aberrant angiogenesis in hypoxic-ischemic retinas. Specifically, a reduced gliotic reaction with lower GFAP expression was noted in Muller cells, while a decrease in VEGF secretion was noted in GCs [25]. The same authors also demonstrated that hypothermia is able to decrease activity of both constitutive NO synthase (nNOS, Ca(2+)-dependent) and inducible NO synthase (iNOS, Ca(2+)-independent) in amacrine, horizontal, and ganglion cells in the context of ischemic proliferative retinopathy [26]. Reinhard et al. demonstrated increased survival time of GCs after retinal artery occlusion in hypothermic conditions. In fact, while action potentials from GCs were no more detected after 4 h from the event at body temperature, a local temperature of 21 °C and 4 °C increased this survival time to 12 h and 50° respectively [27]. 

The effect of hypothermia in counteracting neurodegeneration due to oxidative stress was evaluated by Mueller-Buehl et al. using hydrogen peroxide on retinal cultures. Incubation at 30 °C for 3 h led to preservation of GCs from apoptosis, rescue of amacrine cells and partial protection of bipolar cells. Moreover, microglial response to oxidative stress evaluated 8 days after exposure was completely stopped by hypothermia [28]. Lastly, the previously mentioned TRPM8 channel are expressed in the retina in cholinergic amacrine interneurons and in a subset of melanopsin GCs, where their activation is involved in regulation of cellular action potential [16]. 

#### Vitreous

The vitreous is an acellular transparent hydrogel that occupies two-thirds of the total volume and that is composed of water (98–99%), collagen type II fibrils and hyaluronic acid chains. The latter are primarily responsible for the osmotic pressure that gives the eye its spherical shape and holds the retina in place [29]. The viscoelastic behavior and the transport of molecules in the vitreous is influenced by the binding of water to the macromolecular components. A gradual increase in bound water is present proceeding from the lens to the retina long the optic axis [30]. As a consequence of its composition, vitreous is a thermosensitive material. In fact, bound water, representing 20% of the total water component of the vitreous, and free water are differently affected by temperature changes. In fact, temperature changes in free water occur more rapidly. Moreover, differences in temperature can influence binding properties of vitreous matrix, with higher temperatures leading to weaker binding of water to vitreous macromolecules. Chen et al. documented a lower traumatic effect of posterior vitreous detachment on porcine retinas exposed to 44 °C temperature and then cooled down to room temperature compared to stable room temperature group [31]. The thermosensitive properties of the vitreous served as an inspiration for the synthesis of biomimetic vitreous substitutes. These substances may replace natural vitreous in a more physiological way compared to currently used vitreous substitutes (including silicone oil, saline buffers, expansible gases and perfluorocarbons). Laradji et al. [32] proposed a thermosensitive biomimetic hydrogel that is composed of thiolated gellan (as an analogue of type II collagen) and a polyelectrolyte (as an analogue of hyaluronic acid) that can be injected as a viscous solution at 45 °C in the vitreous cavity. Once in situ, cooling of the hydrogel to body temperature induces disulfide covalent crosslinks formation with gradual transformation of the status of the substitute to a gel with vitreous-like properties.

#### Ciliary body and ocular inflammation

Temperature profoundly affects ciliary body secretory function. Rabbit ciliary body has been shown to experience a critical decrease in hydraulic conductivity and secretory flow when directly exposed to severe hypothermic temperature in vitro [33]. Systemic hypothermia also leads to a dramatic decrease in ciliary body aqueous production. In fact, a rectal temperature of 27 °C induced a reduction of 50% in secretion and a temperature of 20 °C led secretory levels to 10–20% of their normal rate in albino rabbits [34]. At the same time, cooling of ocular temperature has been demonstrated to have protective effects such as reduction of stromal edema of the ciliary body and inhibition of blood–aqueous barrier disruption [35]. In vitro experiments simulating hypoxic conditions on retinal tissue found that hypothermia resulted in a reduction of ROS and stress markers(HSP70,iNOS, HIF-1α), inhibition of apoptotic proteins(caspase 3 and 8) and the cell cycle arrest gene p21, and protection of inner retina neurons and ganglion cells [36–38]. 

### Effects of vitreoretinal surgery on ocular temperature and vitreoretinal surgery performed in hypothermic conditions: state of the art

During vitreoretinal surgery ocular temperature is modulated by additional factors including irrigation and light sources. Measurements of vitreous temperature in human eye at the beginning of vitrectomy (before opening of the infusion line) assessed the presence of a trans-vitreous temperature gradient from the anterior toward the posterior vitreous of the human eye, with the highest temperature recorded in the posterior vitreous. Nevertheless, distribution of intraocular temperature was influenced by the nature of the material filling the vitreous cavity: gradient slope positive inclination was the lowest with BSS (vitreous already removed in a previous surgery), increased in vitreous (first vitrectomy) and significantly increased in silicon oil [39]. During pars plana vitrectomy(PPV), ocular temperature significantly decreases due to infusion of room temperature intraocular fluid and dispersion of choroidal heat due to intraocular flow [40]. As a result, there is a significant decrease in temperatures in vitreous compartments immediately after the beginning of vitrectomy, with the lowest temperature recorded in the anterior vitreous, and the highest temperature decrease compared to baseline, in the preretinal posterior vitreous. These findings were confirmed by Anatychuk et al. [2], that reported a decrease in temperature in all segments of the vitreous cavity during vitreoretinal surgery performed with room temperature irrigation in patients affected by rhegmatogenous retinal detachment(10 eyes) and retinal detachment associated with proliferative diabetic retinopathy(10 eyes). In the absence of continuous irrigation, a rapid rewarming of the vitreous cavity was noted (average 0.18 °C/min). Moreover, manipulation of the vitreous is another factor that may induce increase in ocular temperature during surgery. During vitrectomy, hypothermic conditions seem to reduce iatrogenic damage associated to some routinely performed procedures. Rinkoff et al. [41] demonstrated that exposure to room temperature intraocular fluid (22 °C) prolonged the photochemical and thermal light damage threshold to 60 min, compared to 25 min calculated for body temperature fluid exposure. Coherently, it has also been reported that retinal photochemical and thermal light damage is influenced by body temperature during exposure, even though previous studies do not agree on the magnitude of this effect. An interesting study from Gorgels et al. [42] compared retinal damage of 488 nm wavelength in rats at 30 and 40.5 °C.The dose of radiation needed for a just visible change in fundus decreased by 6% per degree increase in body temperature, with the most damage localizing in the RPE. In 1995, Amara et al. [43] proposed a thermal model of the human eye exposed to laser irradiation allowing us to compute the intraocular temperature distribution. Using this model, the authors investigated the effects of laser power, laser wavelength and other parameters. Among the results obtained, it appeared that the more the laser wavelength decreased the more the effects on the eye were hazardous due to higher temperatures leading to the denaturation of the ocular tissues.

Other authors showed that hypothermia protects cultured human RPE cells against trypan blue toxicity [44]. As concerns postoperative period, regional hyperthermia of the operated eye was demonstrated in 25% of cases [2]. This suggests that vitreoretinal surgery is performed under conditions of uncontrolled local ocular hypothermia and is characterized by a rapid rewarming of the vitreous cavity after cessation of cooling. In a preliminary study on animal models, some authors demonstrated that temperature management, avoidance of intraoperative deep hypothermia, and prevention of rapid uncontrolled rewarming may protect the retinal morphology and increase the safety of prolonged vitreoretinal surgery [45]. As concerns postoperative inflammation, Tamai et al. [35] investigated the effects of irrigation of intraocular spaces with 9 °C, 22 °C and 37 °C solutions for 60 min immediately after experimental vitrectomy in albino rabbits. According to their results, temperature significantly decreased especially at the level of ciliary body and choroid during the following 2 weeks. Parallelly, a decrease in intraocular protein concentration was noted, suggesting a positive effect on intraocular inflammation. The effect of local ocular hypothermia on ocular inflammation was evaluated during experimental open sky vitrectomy, closed vitrectomy, and anterior chamber irrigation and aspiration in 40 albino rabbits (80 eyes) by Jabbour et al. [46] The application of severe local hypothermia (7 °C) was associated to less intraocular bleeding volume, less fibrin production, and less postoperative inflammation. Nevertheless, hypothermia may also have detrimental effects on the eye. In fact, Zilis et al. [47] demonstrated that while intraocular temperatures higher than 22 °C are safely tolerated, irrigation with 2°C may lead to cataract formation and subclinical retinal detachment development. Moreover, cooling of the infusion has been demonstrated to induce electroretinogram changes, whose reversibility in fragile pathologic retina in still a matter of debate [1]. These electroretinography changes were recently analyzed by Romano et al., who compared temperature-controlled PPV and conventional PPV in eyes of ten healthy rabbits. In particular, they revealed that conventional PPV determined a postoperative decreased amplitude and increased latency of a-wave at 3 cd s/m^2^, differently from temperature-controlled one, which also showed an increased amplitude of oscillatory potentials, compared to the decrease after conventional PPV. Finally, immunohistochemistry examination revealed a slight higher value of glial fibrillary acidic protein (GFAP) after standard PPV, in support of the beneficial role of temperature-controlled either in terms of structural and functional results [48]. 

## Conclusions

Temperature changes induce several regulatory responses within the eye, including modifications of IOP, local blood flow, cytokine secretion and cellular metabolism. Cooling of retinal structures has been demonstrated to reduce environmental and iatrogenic damage, especially to RPE and inner retinal layers. Vitreoretinal surgery performed at lower temperatures has shown positive effect on postoperative inflammation, even though the rebound effect of a sudden postoperative temperature increase seems to be detrimental. Despite being a promising approach, vitreoretinal surgery performed under lower intraocular temperature conditions deserves refinement in its methodologies for scientific community to benefit from its positive effects on inflammation and cell preservation. Hopefully, new randomized clinical trials will provide indications on how to apply this technique in the safest and most effective way. A possible strategy would be to obtain stable mild intraoperative hypothermia via compensatory modulation of intraoperative flow and to induce a gradual restoration of preoperative intraocular temperature via a gradual reduction of intraocular flow at the end of surgery. This would allow to perform surgery in a condition of mild hypothermia and to avoid damage from sudden postoperative temperature raise.

## Data Availability

No datasets were generated or analysed during the current study.

## Declarations

### Data sharing statement

The data that support the findings of this study are available from the corresponding author, MMC, upon reasonable request.

## References

[1] Romano MR, Romano V, Mauro A, et al. The effect of temperature changes in vitreoretinal surgery. Translational Vis Sci Technol. 2016;5(1):4. 10.1167/tvst.5.1.4. 10.1167/tvst.5.1.4 PMC4757463 26929884

[2] Anatychuk L, Pasyechnikova N, Naumenko V, et al. Prospects of temperature management in vitreoretinal surgery. Therapeutic Hypothermia Temp Manage. 2021;11(2):117–21. 10.1089/ther.2020.0019. 10.1089/ther.2020.0019 32679001

[3] Polderman KH. Mechanisms of action, physiological effects, and complications of hypothermia. Crit Care Med. 2009;37(7 Suppl):S186–202. 10.1097/CCM.0b013e3181aa5241. 19535947 10.1097/CCM.0b013e3181aa5241

[4] Hou Y, Qiao Y, Xiong M, et al. Hypothermia-rewarming: A Double-edged sword? Med Hypotheses. 2019;133:109387. 10.1016/j.mehy.2019.109387. 31541781 10.1016/j.mehy.2019.109387

[5] Parver LM, Auker C, Carpenter DO. Choroidal blood flow as a heat dissipating mechanism in the macula. Am J Ophthalmol. 1980;89(5):641–6. 10.1016/0002-9394(80)90280-9. 6769334 10.1016/0002-9394(80)90280-9

[6] Heussner N, Holl L, Nowak T, et al. Prediction of temperature and damage in an irradiated human eye-Utilization of a detailed computer model which includes a vectorial blood stream in the choroid. Comput Biol Med. 2014;51:35–43. 10.1016/j.compbiomed.2014.04.021. 24879392 10.1016/j.compbiomed.2014.04.021

[7] Schwartz B, ENVIRONMENTAL TEMPERATURE AND THE OCULAR TEMPERATURE GRADIENT. Arch Ophthalmol. 1965;74:237–43. 10.1001/archopht.1965.00970040239022. 14318502 10.1001/archopht.1965.00970040239022

[8] Kumar S, Acharya S, Beuerman R, Palkama A. Numerical solution of ocular fluid dynamics in a rabbit eye: parametric effects. Ann Biomed Eng. 2006;34(3):530–44. 10.1007/s10439-005-9048-6. 16450187 10.1007/s10439-005-9048-6

[9] Metelitsina TI, Grunwald JE, DuPont JC, et al. Foveolar choroidal circulation and choroidal neovascularization in age-related macular degeneration. Invest Ophthalmol Vis Sci. 2008;49(1):358–63. 10.1167/iovs.07-0526. 18172113 10.1167/iovs.07-0526 PMC3077130

[10] Belmonte C, Garcia-Hirschfeld J, Gallar J. Neurobiology of ocular pain. Prog Retin Eye Res. 1997;16(1):117–56. 10.1016/S1350-9462(96)00027-4.

[11] Reiner A, Fitzgerald MEC, Mar ND, Li C. Neural control of choroidal blood flow. Prog Retin Eye Res. 2018;64:96–130. 10.1016/j.preteyeres.2017.12.001. 29229444 10.1016/j.preteyeres.2017.12.001 PMC5971129

[12] Tamai K, Toumoto E, Yamada K, Ogura Y. Effects of irrigation fluid temperature on choroidal circulation during vitrectomy. Curr Eye Res. 1999;18(4):249–53. 10.1076/ceyr.18.4.249.5361. 10372983 10.1076/ceyr.18.4.249.5361

[13] Horiguchi M, Miyake Y. Effect of temperature on electroretinograph readings during closed vitrectomy in humans. Arch Ophthalmol. 1991;109(8):1127–9. 10.1001/archopht.1991.01080080087035. 1867557 10.1001/archopht.1991.01080080087035

[14] Gallar J, Acosta MC, Belmonte C. Activation of scleral cold thermoreceptors by temperature and blood flow changes. Investig Ophthalmol Vis Sci. 2003;44(2):697–705. 10.1167/iovs.02-0226. 12556401 10.1167/iovs.02-0226

[15] Iovieno A, Lambiase A, Micera A, Stampachiacchiere B, Sgrulletta R, Bonini S. In vivo characterization of Doxycycline effects on tear metalloproteinases in patients with chronic blepharitis. Eur J Ophthalmol. 2009;19(5):708–16. 10.1177/112067210901900504. 19787586 10.1177/112067210901900504

[16] Reimúndez A, Fernández-Peña C, Ordás P, et al. The cold-sensing ion channel TRPM8 regulates central and peripheral clockwork and the circadian oscillations of body temperature. Acta Physiol. 2023;237(3):e13896. 10.1111/apha.13896. 10.1111/apha.13896 36251565

[17] Osborne NN, Casson RJ, Wood JPM, et al. Retinal ischemia: mechanisms of damage and potential therapeutic strategies. Prog Retin Eye Res. 2004;23(1):91–147. 10.1016/j.preteyeres.2003.12.001. 14766318 10.1016/j.preteyeres.2003.12.001

[18] Shweiki D, Itin A, Soffer D, Keshet E. Vascular endothelial growth factor induced by hypoxia May mediate hypoxia-initiated angiogenesis. Nature. 1992;359(6398):843–5. 10.1038/359843a0. 1279431 10.1038/359843a0

[19] Coassin M, Duncan KG, Bailey KR, et al. Hypothermia reduces secretion of vascular endothelial growth factor by cultured retinal pigment epithelial cells. Br J Ophthalmol Published Online January. 2010;1. 10.1136/bjo.2009.168864. 10.1136/bjo.2009.168864 20805126

[20] Takeyama M, Yoneda M, Gosho M, et al. Decreased VEGF-A and sustained PEDF expression in a human retinal pigment epithelium cell line cultured under hypothermia. Biol Res. 2015;48(1):42. 10.1186/s40659-015-0034-7. 26223306 10.1186/s40659-015-0034-7 PMC4518530

[21] Larrayoz IM, Rey-Funes M, Contartese DS, et al. Cold shock proteins expression in the retina following exposure to hypothermia. Investig Ophthalmol Vis Sci. 2017;58(8):3592.28715845

[22] Sun YJ, Zhang ZY, Fan B, Li GY. Neuroprotection by Therapeutic Hypothermia. *Frontiers in Neuroscience*. 2019;13. Accessed February 11, 2024. https://www.frontiersin.org/journals/neuroscience/articles/10.3389/fnins.2019.00586 10.3389/fnins.2019.00586 PMC6579927 31244597

[23] Mandai M, Watanabe A, Kurimoto Y, et al. Autologous induced Stem-Cell–Derived retinal cells for macular degeneration. N Engl J Med. 2017;376(11):1038–46. 10.1056/NEJMoa1608368. 28296613 10.1056/NEJMoa1608368

[24] Kitahata S, Tanaka Y, Hori K, et al. Critical functionality effects from storage temperature on human induced pluripotent stem cell-Derived retinal pigment epithelium cell suspensions. Sci Rep. 2019;9(1):2891. 10.1038/s41598-018-38065-6. 30814559 10.1038/s41598-018-38065-6 PMC6393435

[25] Rey-Funes M, Dorfman VB, Ibarra ME, et al. Hypothermia prevents gliosis and angiogenesis development in an experimental model of ischemic proliferative retinopathy. Invest Ophthalmol Vis Sci. 2013;54(4):2836–46. 10.1167/iovs.12-11198. 23471892 10.1167/iovs.12-11198

[26] Rey-Funes M, Ibarra ME, Dorfman VB, et al. Hypothermia prevents nitric oxide system changes in retina induced by severe perinatal asphyxia. J Neurosci Res. 2011;89(5):729–43. 10.1002/jnr.22556. 21337363 10.1002/jnr.22556

[27] Reinhard K, Mutter M, Gustafsson E, et al. Hypothermia promotes survival of ischemic retinal ganglion cells. Invest Ophthalmol Vis Sci. 2016;57(2):658–63. 10.1167/iovs.15-17751. 26903226 10.1167/iovs.15-17751

[28] Mueller-Buehl AM, Doepper H, Grauthoff S, et al. Oxidative stress-induced retinal damage is prevented by mild hypothermia in an ex vivo model of cultivated Porcine retinas. Clin Exp Ophthalmol. 2020;48(5):666–81. 10.1111/ceo.13731. 32077190 10.1111/ceo.13731

[29] Swindle KE, Ravi N. Recent advances in polymeric vitreous substitutes. Expert Rev Ophthalmol. 2007;2(2):255–65. 10.1586/17469899.2.2.255.

[30] Ali S, Bettelheim FA. Distribution of freezable and non-freezable water in bovine vitreous. Curr Eye Res. 1984;3(10):1233–9. 10.3109/02713688409000827. 6488852 10.3109/02713688409000827

[31] Chen K, Weiland JD. Relationship between vitreous temperature and posterior vitreous detachment. J Mech Behav Biomed Mater. 2013;26:54–8. 10.1016/j.jmbbm.2013.05.015. 23816597 10.1016/j.jmbbm.2013.05.015

[32] Laradji A, Shui YB, Karakocak BB, et al. Bioinspired thermosensitive hydrogel as a vitreous substitute: synthesis, properties, and progress of animal studies. Mater (Basel). 2020;13(6):1337. 10.3390/ma13061337. 10.3390/ma13061337 PMC7143394 32183465

[33] Green K, Pederson J. Effect of temperature on water flow through the ciliary epithelium. Am J Physiology-Legacy Content. 1972;222(5):1227–9. 10.1152/ajplegacy.1972.222.5.1227. 10.1152/ajplegacy.1972.222.5.1227 5022380

[34] Holmberg Å, Becker B. The effect of hypothermia on aqueous humor dynamics**. Am J Ophthalmol. 1960;49(5, Part 2):113448–4054. 10.1016/0002-9394(60)91625-1.

[35] Tamai K, Toumoto E, Majima A. Protective effects of local hypothermia in vitrectomy under fluctuating intraocular pressure. Exp Eye Res. 1997;65(6):733–8. 10.1006/exer.1997.0386. 9441696 10.1006/exer.1997.0386

[36] Schultheiss M, Schnichels S, Hermann T, et al. Hypothermia protects and prolongs the tolerance time of retinal ganglion cells against ischemia. PLoS ONE. 2016;11(2):e0148616. 10.1371/journal.pone.0148616. 26848953 10.1371/journal.pone.0148616 PMC4744055

[37] Salido EM, Dorfman D, Bordone M, et al. Global and ocular hypothermic preconditioning protect the rat retina from ischemic damage. PLoS ONE. 2013;8(4):e61656. 10.1371/journal.pone.0061656. 23626711 10.1371/journal.pone.0061656 PMC3633982

[38] Maliha AM, Kuehn S, Hurst J, et al. Diminished apoptosis in hypoxic Porcine retina explant cultures through hypothermia. Sci Rep. 2019;9(1):4898. 10.1038/s41598-019-41113-4. 30894574 10.1038/s41598-019-41113-4 PMC6427006

[39] Shinoda K, Yagura K, Matsumoto S, et al. Intraocular temperature at different sites in eye measured at the beginning of vitreous surgery. J Clin Med. 2021;10(15):3412. 10.3390/jcm10153412. 34362195 10.3390/jcm10153412 PMC8348370

[40] Landers MB, Watson JS, Ulrich JN, Quiroz-Mercado H. Determination of retinal and vitreous temperature in vitrectomy. Retina. 2012;32(1):172–6. 10.1097/IAE.0b013e31821c3ee0. 21878844 10.1097/IAE.0b013e31821c3ee0

[41] Rinkoff J, Machemer R, Hida T, Chandler D. Temperature-Dependent light damage to the retina. Am J Ophthalmol. 1986;102(4):452–62. 10.1016/0002-9394(86)90073-5. 3766660 10.1016/0002-9394(86)90073-5

[42] Gorgels TGMF, van Beek L, van Norren D. Effect of body temperature on retinal damage by 488 Nm light in rat. Microsc Res Tech. 1997;36(2):89–95. 10.1002/(SICI)1097-0029(19970115)36:2<89::AID-JEMT2>3.0.CO;2-Q. 9015255

[43] Amara EH. Numerical investigations on thermal effects of laser-ocular media interaction. Int J Heat Mass Transf. 1995;38(13):2479–88. 10.1016/0017-9310(94)00353-W.

[44] Kunikata H, Tomita H, Murata H, et al. Hypothermia protects cultured human retinal pigment epithelial cells against Trypan blue toxicity. Ophthalmologica. 2006;220(2):114–7. 10.1159/000090576. 16491034 10.1159/000090576

[45] Anatychuk L, Zadorozhnyy O, Naumenko V, et al. Vitreoretinal surgery with temperature management: A preliminary study in rabbits. Therapeutic Hypothermia Temp Manage. 2023;13(3):126–33. 10.1089/ther.2022.0044. 10.1089/ther.2022.0044 36827431

[46] Jabbour NM, Schepens CL, Buzney SM. Local ocular hypothermia in experimental intraocular surgery. Ophthalmology. 1988;95(12):1687–90. 10.1016/s0161-6420(88)32956-8. 3231436 10.1016/s0161-6420(88)32956-8

[47] Zilis JD, Chandler D, Machemer R. Clinical and histologic effects of extreme intraocular hypothermia. Am J Ophthalmol. 1990;109(4):469–73. 10.1016/S0002-9394(14)74615-X. 2330950 10.1016/s0002-9394(14)74615-x

[48] Romano MR, Barachetti L, Ferrara M, Mauro A, Crepaldi L, Bronzo V, Franzo G, Ravasio G, Giudice C. Temperature control during pars plana vitrectomy. Graefes Arch Clin Exp Ophthalmol. 2024 Sep 9. 10.1007/s00417-024-06631-6. Epub ahead of print. PMID: 39249514. 10.1007/s00417-024-06631-6 39249514

